# Isolated acetabular cup revision in Metal-on-Metal total hip arthroplasty: a low-complication strategy feasible in only half of cases

**DOI:** 10.1007/s00264-025-06534-z

**Published:** 2025-04-21

**Authors:** Cristobal Duda, Pierre-Alban Bouché, Morgan Gauthier, Amanda Gonzalez, Matthieu Zingg, Didier Hannouche

**Affiliations:** 1 University Hospital of Geneva, Geneva, Switzerland; 2 University of Geneva, Geneva, Switzerland; 3 Université Paris Cité, Paris, France

**Keywords:** Total hip arthroplasty, Revision, Metal-on-Metal, ALTR

## Abstract

**Purpose:**

There is still a debate regarding the removal of the femoral stem due to the risk of trunnion. To answer this question, we conducted a study to compare long terms outcomes of isolated acetabular to total revision of MoM THA using an institutional arthroplasty registry.

**Methods:**

From 1996 to 2019, 150 patients (12.5%) of the 1202 revision THAs (rTHA) recorded in Geneva Arthroplasty Registry (GAR) underwent a revision of a MoM THA. After matching the two groups,126 patients were finally included: 63 in each group. The mean age was 64.4 (SD 11.6) years, 48.4% (61/126) were women with a mean BMI of 27.2 (SD 5.5) Kg/m2.

**Results:**

The overall survival rate was 88.1% [79.9–97.2%] at ten years. 10-year survival rate was 93.5% [86.2–100.0%] after isolated acetabular rTHA and 79.5% [61.7–100.0%] after total rTHA (*p* = 0.16). Regarding Hip Harris score and Merle d’Aubigne score, no difference at last follow-up was observed between the two groups (respectively: *p* = 0.39; *p* = 0.33). Regarding the chrome, cobalt, and nickel level reduction, no difference was observed between the two groups (respectively, *p* = 0.38, 0.81 and 0.97).

**Conclusion:**

No difference was observed between isolated acetabular and total revision of MoM THAs regarding survival rate and ions levels at long term. It seems advisable to perform an isolated acetabular revision of a MoM THA when it is indicated.

**Levels of evidence:**

Level III, case control studies.

**Supplementary Information:**

The online version contains supplementary material available at 10.1007/s00264-025-06534-z.

## Introduction

The theoretical advantages of Metal-on-Metal (MoM) bearings include superior wear behavior and the ability to use large-diameter femoral head components to minimize the risk of dislocation after surgery [1, 2].

Unfortunately, the failure rates of MoM implants have been higher than expected [3], as evidenced in registries, with revision rates as high as 5–10% at 5 years. This was mainly due to implant loosening and/or osteolysis, and hypersensitivity to metal ions released by the prosthesis [4–6]. The most common indications for revision of MoM Total Hip Arthroplasty (THA) include adverse local tissue reaction (ALTR), aseptic loosening of the acetabular component, infection, and persistent groin pain [7].

Some studies have suggested that the soft tissue damage resulting from the failure of primary MoM hip components may also predispose patients to higher rates of complications after revision surgery, including dislocation and infection, especially after isolated revisions of the acetabular component [8, 9]. In a recent study, Jennings et al. [10] showed that patients who underwent isolated modular MoM head-liner exchange faced higher complication rates and a higher risk of re-revision surgery, as compared to patients undergoing acetabular-only or all-component revision (acetabular and femoral).

Isolated acetabular revisions might be challenging and raise some concerns over potential complications arising from stem retention. However, they also have potential advantages over both-component revisions, including decreased blood loss and reduced surgical time [11].

To our knowledge, no study has compared the long-term outcome of isolated acetabular revision with total (both components) revision in MoM THA.

Therefore, the aim of this study was to compare the overall survival rate of isolated acetabular to total revision of MoM THA using prospectively collected data from the Geneva Arthroplasty Registry (GAR). The long-term clinical scores, rate of complications and the effect of the revision on the serum metal ions levels were assessed as secondary outcomes. Finally, we tried to identify risk factors of failure in revision THA (rTHA) after MoM bearing.

## Patients and methods

The local ethics committee (N°: PB_2017 − 00164) approved this single-center, prospective cohort; all patients gave informed consent to participate.

### Patients

**Fig. 1:**
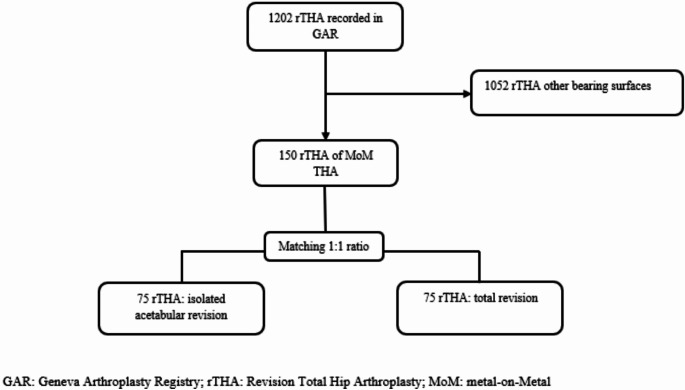
Flow chart

Since 1996, all patients undergoing THA at Geneva University Hospitals have been enrolled in the GAR. Up to 2019, 150 patients (12.5%) over the 1202 rTHA recorded in GAR had a revision of a MoM THA. Seventy-five patients (50%) had an isolated acetabular revision. At our institution, an isolated acetabular revision of MoM THA was proposed in the following situations: (i) ALTR with or without osteolysis; (ii) aseptic loosening of the acetabular component without radiologic or intraoperative sign of femoral loosening; (iii) persistent groin pain, with elevation of systemic levels of chromium (Cr) and cobalt (Co) ions in whole blood (within the range of 2 to 7 µg/L, according to the EFORT consensus statement [12]) and/or presence of osseous and soft-tissue abnormalities such as synovitis, joint effusion, acetabular osteolysis, intramuscular edema identified on MRI sequences without radiologic sign of femoral loosening [13]; (iv) iliopsoas impingement; and (v) recurrent dislocation. A total rTHA was chosen in the following situations: (i) a loosening, migration of the femoral component, or femoral osteolysis; (ii) intraoperative macroscopic signs of trunnionosis at the femoral neck. After matching isolated acetabular and total rTHA by propensity score, 126 patients were finally included: 63 in each group (Fig. 1). The main cause of revision was ALTR in 69.8% (88/126) followed by aseptic loosening (15.1%,19/126). The mean age of the population was 64.4 (SD 11.6) years, 48.4% (61/126) of the patients were women, with a mean body mass index (BMI) of 27.2 (SD-5.5) Kg/m2. Sixty-two patients (49.2%) had been treated with large-head (diameter ≥ 34 mm) MoM THA and 64 patients (50.8%) with a small-head (diameter < 34 mm) at the index operation. The mean time to revision was 7.8 (SD 3.9) years. The mean follow-up after revision was 4.0 years (SD 3.1). Demographic data in the two groups are presented in Table 1.

**Table 1:** Demographic data

Parameters	Values	*n*	Isolated acetabular rTHA	*n*	Total rTHA	*p*-value
Age (years)		63	63.9(10.34)	63	64.9(12.7)	0.92
BMI (kg/m^2^)		63	27.2(5.871)	63	27.3(5.2)	0.73
Gender	Female	31	49.2%	30	47.6%	1.00
	Male	32	50.8%	33	52.4%	
ASA score		63	2.0(0.5)	63	1.9(0.6)	0.41
Tobacco	No	43	68.3%	50	79.4%	0.22
	Yes	20	31.7%	13	20.6%	
Time to revision (years)		63	7.2 (3.9)	62	8.4 (3.8)	0.09
Cause of revision	ALTR	47	79.7%	41	65.1%	< 0.01
	Aseptic loosening	6	10.2%	13	20.6%	
	Groin pain	1	1.7%	0	0.0%	
	Iliopsos impigement	4	6.8%	0	0.0%	
	Instability	1	1.7%	0	0.0%	
	Peri-prosthetic fracture	0	0.0%	9	14.3%	
Paprosky classfication for acetabular defects	1	48	77.4%	53	84.1%	0.53
	2 A	9	14.5%	5	7.9%	
	2B	2	3.2%	4	6.3%	
	2 C	2	3.2%	1	1.6%	
	3B	1	1.6%	0	0.0%	
Diameter of femoral metal head	Large (≥ 34 mm)	27	36.0%	46	61.3%	< 0.01
	Small (< 34 mm)	48	64.0%	29	38.7%	
Mean time to revision (years)		63	7.3(3.9)	62	8.4(3.8)	0.09

ASA : American Society Anesthesiologists

### Surgical data

The modular femoral head was replaced in all revisions. Four surgical approaches were performed: direct anterior in 4.0% (5/126), lateral approach in 11.0% (14/126), posterior approach in 81.0% (102/126) and transtrochanteric in 4.0% (5/126). Four types of bearings were used: ceramic-on-polyethylene in 1.6% (2/126), ceramic-on- polyethylene HXL in 85.7% (108/126), metal-on-metal in 3.2% (4/126) and metal-on-polyethylene in 9.5% (12/126). In the isolated acetabular rTHA, all initial femoral heads were replaced on the old Morse through a sleeve with a metal head in 11 cases (17.5%) and ceramic heads in 82.5% (52/63).A dual mobility cup was used in 58.7% (74/126) and acetabular cup reinforcement in 27.0% (34/126). Surgical data in the two groups are presented in Table 2.

**Table 2:** Surgical data

Parameters	Values	*n*	Isolated acetabular rTHA	*n*	Total rTHA	*p*-value
Surgical approaches	Anterior Hueter	4	6.30%	1	1.60%	0.16
	Transgluteal	10	15.90%	4	6.30%	
	Posterior	46	73%	56	88.90%	
	Trochanteric flip	3	4.80%	2	3.20%	
Bearings surface	Ceramic-polyethylene	1	1.60%	1	1.60%	0.46
	Ceramic-polyethylene HXL	51	81%	57	90.50%	
	Metal-metal	3	4.80%	1	1.60%	
	Metal-polyethylene	8	12.70%	4	6.30%	
Dual mobility cup	Yes	42	66.70%	32	50.80%	0.1
	No	21	33.30%	31	49.20%	
Acetabular cup reinforcement	Yes	20	31.70%	14	22.20%	0.32
	No	43	68.30%	49	77.80%	

Post-operative rehabilitation included early mobilization and restriction to 50% weight bearing with two crutches until the sixth post-operative week.

### Outcomes

Demographic data including age, sex, BMI and American Society of Anesthesiologists (ASA) physical status were recorded for each patient on the day of surgery. Clinical follow-up of each patient was recorded in the GAR at one, five, ten, 15 and 20 years after surgery. At final follow-up, Harris Hip Score (HHS) and Merle d’Aubigné Score (MA), physical examination and radiographic control were performed. Data on surgical revision and surgical complications were available for all patients. Serum samples were collected and analyzed to assess serum Cobalt (Co) and Chromium (Cr) levels. The serum Co and Cr ions levels were measured before and after revision, within 12 months of revision, to determine whether serum level fluctuations differed among patients who experienced complications or not.

### Statistics

To match the two groups and limit the impact of external factors as well as any selection bias, propensity score matching was used. The propensity score was calculated by considering the patient’s age at time of surgery, sex, BMI and ASA score. Each patient who underwent a unipolar rTHA was matched with a patient that underwent a total rTHA on a 1:1 basis. Matching was performed with a logit scale using a 0.3 caliper. Frequencies and percentages were used to describe discrete variables, continuous variables were described using means and standard deviations. For comparative analyses, the Fischer exact test was used for percentages and the Wilcoxon test for continuous variables. Kaplan Meier analysis was performed to analyze survival rate, defined as reoperation for any cause, at the last follow-up. The association of risk factors with failure rate was evaluated using univariate Cox proportional hazards regression models. Hazard ratios (HRs) are reported with the 95% CI. *P* ≤ 0.05 was considered to be significant, for a power of 80% and an alpha risk of 5%. R software (version 3.5.0) was used to perform statistical analyses (https://www.r-project).

## Results

### Survival analysis

The overall survival rate in our series of MoM rTHA was 88.1% [79.9-97.2%] at ten years.

In isolated acetabular rTHA, 4.8% (3/63) had a re-revision at the last follow-up: two for recurrent dislocations (66.6%,2/3) and one for aseptic loosening of the acetabular cup (33.4%,1/3). The overall survival rate in this group was 98.4% [95.3-100.0%] at one year, 93.5% [86.2-100.0%] at five years and 93.5% [86.2-100.0%] at ten years.

**Fig. 2:**
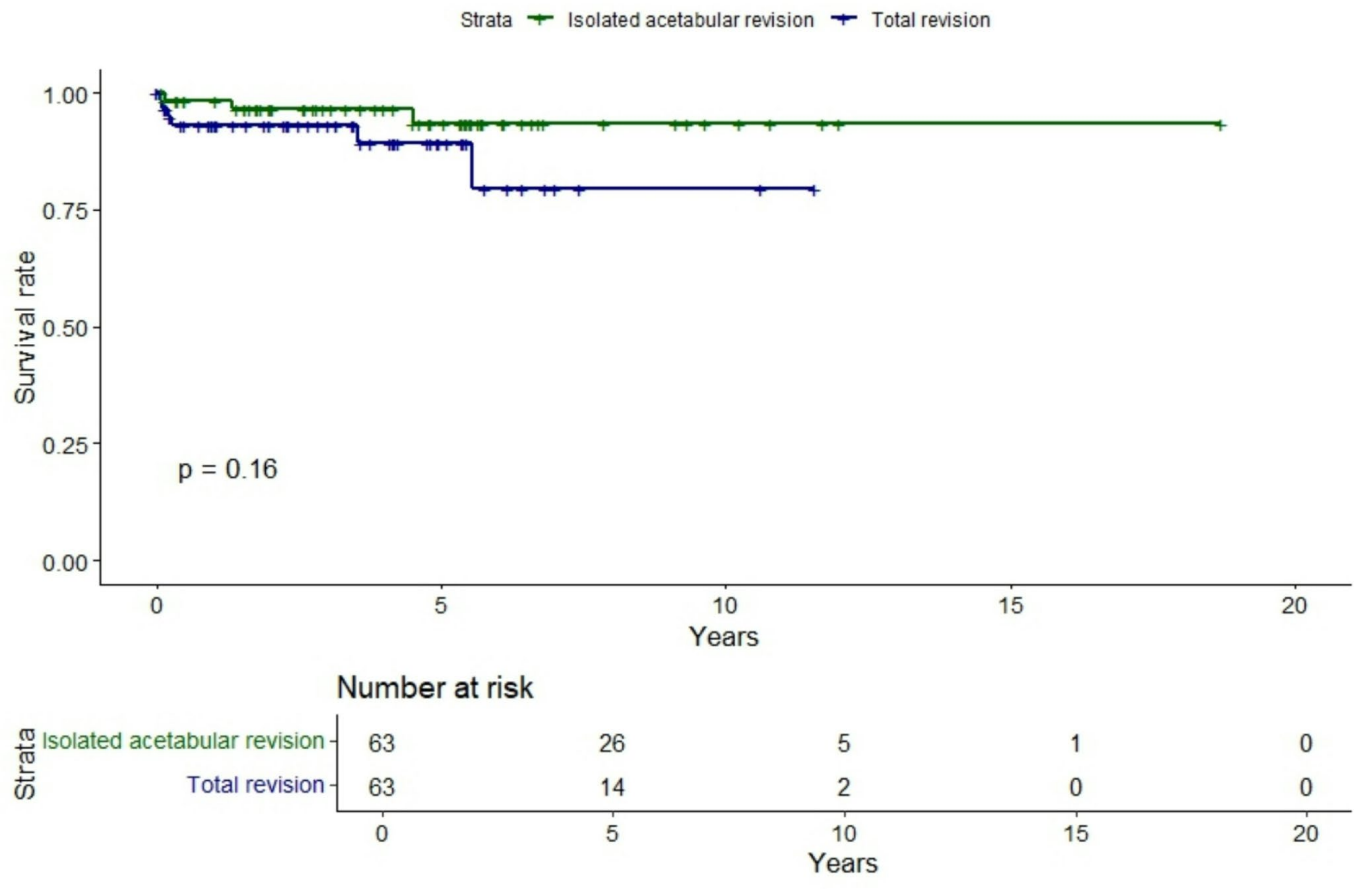
Comparison of revision rate between ONA and OA THA

In total rTHA, 9.5% (6/63) had a re-revision at the last follow-up: four for recurrent dislocation (66.6%,4/6), one for aseptic loosening of femoral stem (16.7%,1/6) and one for recurrent cyst formation (16.7%,1/6). The overall survival rate in this group was 93.0% [86.6-99.9%] at one year, 89.4% [80.5-94.5%] at five years and 79.5% [61.7-100.0%] at ten years. No significant differences were observed between the groups at each endpoint (*p* = 0.16, Fig. 2).

### Clinical outcomes

Regarding the pain subscale of HHS, HHS and MA score, no difference at last follow-up was observed between the two groups (respectively: *p* = 0.19; *p* = 0.39; *p* = 0.33, Table 3). Regarding hemoglobin level, blood loss was higher in total rTHA (4.0 g/dL+/-1.3)) compared to isolated acetabular rTHA (3.2 g/dL+/-12.5); *p* < 0.01).

**Table 3:** Clinical and biological outcomes

Parameters	*n*		Isolated acetabular rTHA		*n*	Total rTHA	*p*-value
**Pain subscale of Harris score**							
Pre-operative	51			22.8 (12.4)	33	21.9 (12.1)	0.58
Last follow-up	34			38.6 (8.6)	26	34.0 (12.1)	0.19
Difference	31			15.9 (15.2)	18	12.4 (14.8)	0.23
**Harris score**							
Pre-operative		38		64.9 (21.9)	24	65.4 (21.0)	0.80
Last follow-up		33		87.5 (16.8)	23	80.7 (23.3)	0.39
Difference		20		22.9 (26.6)	11	19.6 (23.7)	0.66
**Merle d’Aubigne score**							
Pre-operative	56			14.0 (2.8)	45	14.3 (2.7)	0.58
Last follow-up	55			16.5 (1.5)	49	16.5 (2.1)	0.33
Difference	50			2.4 (2.6)	39	2.6 (2.9)	0.14
**Haemoglobin level (g/dL)**							
Pre-operative		63		14.0 (13.6)	63	13.7 (13.8)	0.24
Last follow-up		63		10.8 (14.0)	63	9.6 (13.6)	**< 0.01**
Difference		63		3.2 (12.5)	63	4.0 (1.3)	**< 0.01**
**Chrome level (nmol/L)**							
Pre-operative		45		72.1 (53.1)	43	71.9 (57.2)	0.97
Last follow-up		31		40.0 (38.5)	25	56.1 (31.2)	**0.01**
Difference		31		38.0 (41.0)	36	36.2 (56.1)	0.38
**Cobalt level (nmol/L)**							
Pre-operative	45			94.2 (96.1)	43	76.4 (98.4)	0.60
Last follow-up	32			42.5 (60.5)	25	41.0 (44.1)	0.73
Difference	32			57.4 (74.9)	25	68.0 (101.1)	0.81
**Nickel level (nmol/L)**							
Pre-operative	24			26.6 (33.1)	21	22.7 (12.8)	0.59
Last follow-up	22			14.1 (7.7)	14	17.5 (7.4)	0.11
Difference	14			16.7 (42.1)	9	7.8 (15.7)	0.97

A total of eleven complications (8.7%, 11/126)) were observed: two cases of aseptic loosening (1.6%, 2/126) and nine dislocations (7.1%, 9/126). Four complications were reported after isolated acetabular rTHA (6.3%,4/63): one aseptic loosening (25%, 1/4) and three dislocations (75%, 3/4). Seven complications were reported in total rTHA (11.1%,7/63): one aseptic loosening (14.3%, 1/7) and six dislocations (85,7%, 6/7). No differences were observed between groups regarding the rate of complications (*p* = 0.53) and the causes of complications (*p* = 1.00).

Regarding chrome level at last follow-up, a significant difference was observed with a higher level in total rTHA group (56.1 nmol/L+/-31.2)) compared to isolated acetabular rTHA (40.0nmol/L+/-38.5); *p* = 0.01). This difference wasn’t observed regarding difference between preoperatively and at last follow-up chrome level (Table 3).

Regarding cobalt and nickel level, no difference was observed between groups (Table 3).

### Risk factors

The univariate analysis included age at the surgery, gender, BMI, ASA score, tobacco status, use of dual mobility cup, use of acetabular cup reinforcement, chrome, cobalt, and nickel level preoperative. No failure risk factor was identified (Table 4).

**Table 4:** Risk factors of failure after rTHA of mom

Characteristic	HR^1^	95% CI^1^	*p*-value
Age	1.01	0.95;1.07	0.69
BMI	1.03	0.92;1.15	0.57
Gender			
Women	-	-	
Men	3.26	0.68;15.70	0.14
ASA	1.00	0.28;3.59	0.99
Tobacco	-	-	
No	0.74	0.15;3.56	0.71
Yes			
Dual mobility cup	1.09	0.88,1.36	0.42
Yes	-	-	
No	2.70	0.68;10.83	0.16
Acetabular cup reinforcement			
No	-	-	
Yes	0.72	0.15;3.48	0.69
Femoral head size preoperative			
Large	-	-	
Small	0.90	024 ;3.37	0.88
Chrome level preoperative	0.99	0.97 ;1.02	0.66
Cobalt level preoperative	0.99	0.98 ;1.01	0.36
Nickel level preoperative	-	-	

^1^ HR = Hazard Ratio, CI = Confidence Interval

## Discussion

Causes for revisions in MoM THA have differed from those previously observed in THA using others bearing surfaces [14–18]. The main cause of revision with MoM bearings is ALTR [7, 19]. MoM bearings can release corrosion and fretting debris into the local soft-tissue environment triggering a periprosthetic inflammatory response [20]. This reaction can cause deep and invasive necrosis in soft-tissues [21, 22], resulting in pain, cysts, osteolysis and muscle damage [23, 24].

In ALTR revision, the main goal of surgery is to change the MoM couple and replace it with another combination of bearing surface [25]. However, there is a significant debate regarding the femoral stem: should it be retained or revised, considering the potential risk of damage to the trunnion [5, 26–28]. Removing a well-fixed femoral stem can increase the risk of complications, including bone loss, femoral fracture, soft tissue damage, prolonged recovery and failure to achieve fixation [7]. To address this critical question, we conducted a study comparing the overall survival rate and the rate of complications between isolated acetabular and total revision of MoM THA.

The overall survival free of re-revision, in our series, was 88.1% [79.9-97.2%] at ten years. Goldman et al. [29] reported the survival rate of 2589 aseptic revision THAs including all types of bearings. They found an overall survival rate of 88% at ten years. Erden et al. [30] included 80 revisions due to trunnion corrosion in primary metal-on-polyethylene THA and found, at five years, a global survival rate at 73.2%. Matharu et al. [31] reported a ten-year reoperation-free survival rate of 63% for revised MoM hip resurfacing, with 45% (24 of 53) experiencing complications and 38% (20 of 53) undergoing reoperation. Compared to the literature, our study shows that performing a revision after a MoM primary THA does not seem to increase the risk of re-revision. Furthermore, we did not find a significant difference between isolated acetabular revision or total rTHA between survival rates, although there was a trend in favour of isolated acetabular revision at ten years (93.5% for isolated revision Vs. 79.5% for total revision). In a series of isolated revision of an acetabular component to a ceramic-on-ceramic bearing in patients under 50 years of age, the survivorship was 93% at 15 years [32]. Fishley et al. [33] compared isolated femoral revisions with total revision after MoM hip arthroplasty. They reported a five year survival rate of 90.0% for isolated femoral revision and 95.9% for total revision, with no significant difference (*p* = 0.248).

Our complication rate was 8.7% (loosening, fracture, dislocation), which is low in comparison with other studies. Munro et al. [5]have demonstrated a high rate of complications (38%) after revision of large-head MoM in THA in a series of 32 hips. Dislocation rate was high (28%) and was the main cause of revision. Meriem et al. [34] reported 17% of complications (11/64) after revision of large-head MoM in THA with dual cup of mobility with five dislocations (5/64, 8%). The most common complication in our study was also dislocation but with a lower rate of 7.1%. This may be explained by the fact that 58.7% of our patients had a dual mobility cup [35]. Bonner et al. [24] have experienced a high level of re-revision (16% in a series of 40 hips) after a MoM THA revision, which was mostly due to dislocation in 38% of the patients. In this study, 80% had a dual mobility cup or a constrained construct. It is important to precise that their majority of patients had a total revision and not just a single cup revision. Stryker et al. [36] showed a high early complication rate after revision of monoblock MoM THAs. 20% of revisions involved, at least, one early complication after surgery with 16% undergoing at least one additional subsequent surgery. The most common complication was aseptic loosening (6%) and deep infection (6%) with a follow-up of five years. No factors were incriminated in this study to stratify the risk of reoperation. In our study, we had a rate of 1.6% of aseptic loosening: They all had a reoperation. We, only, observed one recurrence of a cyst, but this was in the total rTHA group (0.8%). Interestingly, the results of the present study indicate that isolated revision of the acetabular component in MoM THAs is a viable option, and that there is no need to change the stem preventively to avoid the risk of trunnion damage in the long term. Our study showed a low rate of complications including infection requiring reoperation. One study showed a high risk of complications, dislocation and reoperation in patients with a modular head-liner exchange revision MoM THA at short-term followup [9] with a total of 15 of 54 (28%) complications, including 12 of 54 (22%) patients with dislocation and three with deep infection (6%).The rate of complications can be associated with the reason of revision like exposed in the study by Matharu et al. [37].

Regarding, functional outcomes at the last follow-up, no difference was observed between isolated or total rTHA. We noticed, regarding the delta between pre- and post-operative scores, an improvement of HHS (22.9 and 19.6 increase, respectively in isolated and total revision, Table 2) and MA score (2.4 and 2.6 increase, respectively in isolated and total revision) at a mean follow-up of four years (Table 2). Persson and al. [38] reported similar results on increasing the HHS, at one year, after revision for symptomatic pseudotumour after metal-on-polyethylene THA with a standard femoral stem. Concerning the rate of metal ions, a decrease in blood levels between the pre and postoperative revision levels was observed. However, this difference was not significant between isolated cup revision group and total revision group. Following cobalt-chromium-molybdenum (CoCrMo) to alumina ceramic head replacement, Plummer et al. [39] reported post-operative metal ion levels at a mean of 2.7 years follow-up with a significant reduction in serum cobalt levels over time from 11.6 ppb to 0.3 ppb. Fishley et al. [33], comparing isolated femoral revisions with total revision after MoM THA, found a global significant reduction in serum metal ions postoperatively (*p* < 0.001) and no difference between groups (cobalt: *p* = 0.674);chromium: *p* = 0.186).

Our study has several limitations. Firstly, as a no randomized trial, it is susceptible to all the flaws inherent to this study design. The potential for selection bias exists because there was not a uniform criterion for a revision diagnosis of ALTR. Rather, the diagnosis was made when other potential diagnoses were excluded and a constellation of findings including ion levels, patient symptoms, component position, high-risk implant type, and advanced imaging supported the diagnosis. Although it is not a randomized controlled trial, we obtained two comparable groups for age at time of surgery, sex, BMI and ASA score using propensity score. It can therefore be considered a pseudo-randomized study. Secondly, the use of revision implants was not standardized. At the time, our centre did not have a standardized protocol for MoM revision. Also, the limited follow-up in our study presents a weakness. Certainly, longer follow-up will identify additional failures, but it must be noted all identified complications occurred within five years of the index revision. Finally, although it represents the largest series which compared isolated revision of acetabular component and total rTHA of MoM THA to date, the number of events per group is relatively small limiting the ability to detect significant differences in survival and complication rates.

## Conclusion

No difference was observed in the survival rate and ions levels between isolated acetabular revision and total revision of MoM THA over the long term. Additionally, at a mean follow-up of four years, no new cases of ALTR were observed following isolated acetabular revision. These findings suggest that isolated acetabular revision of MoM THA is a low complication strategy but is only feasible in half of the cases. It will be valuable to compare the outcomes of isolated acetabular revision of MoM THA with those of other bearings, such as ceramic- on-ceramic or metal-on-polyethylene THA.

## Electronic supplementary material

Below is the link to the electronic supplementary material.

Supplementary Material 1

## Data Availability

No datasets were generated or analysed during the current study.
